# A mixed methods study of non-occupational post-exposure prophylaxis at an STI clinic in Singapore: Five-year retrospective analysis and providers' perspectives

**DOI:** 10.1371/journal.pone.0202267

**Published:** 2018-08-20

**Authors:** Alvin Kuo Jing Teo, Bee Choo Tai, Martin Tze-Wei Chio, Hanh Hao La

**Affiliations:** 1 Saw Swee Hock School of Public Health, National University of Singapore and National University Health System, Singapore, Singapore; 2 Department of Sexually Transmitted Infections Control, National Skin Centre, Singapore, Singapore; University of Washington, UNITED STATES

## Abstract

**Background:**

This mixed methods study aims to describe 1) characteristics of the population treated with non-occupational post-exposure prophylaxis (nPEP), 2) predictors of loss to follow-up (LTFU) and nPEP adherence, and 3) to evaluate the nPEP prescribing practices against current management guideline.

**Methods:**

This study was conducted at the Department of Sexually Transmitted Infections Control Clinic in Singapore using clinical data from 2010 to 2016. Explanatory sequential mixed method design was adopted. Predictors of LTFU and nPEP adherence were assessed using modified Poisson regression with robust sandwich variance. Subsequently, nine in-depth interviews with healthcare providers were conducted to gain their insights into barriers and facilitators to nPEP implementation. Transcripts were coded and themes were explored using applied thematic analysis.

**Results:**

Of 502 nPEP cases reviewed, 46% were LTFU, 42% were adherent to nPEP and 431 prescription decisions were made in accordance with the guideline. Tourists (aRR, 2.29 [1.90–2.74]; *p*<0.001) and men who have sex with men/bisexual men (aRR, 1.32 [1.09–1.59]; *p* = 0.004) were significant predictors of LTFU. Absence of side effects (aRR, 1.14 [1.02–1.27]; *p* = 0.024) and nPEP treatment with TDF/FTC/ATV/r (aRR, 1.15 [1.03–1.29]; *p* = 0.017) were positively associated with nPEP adherence. Stigma, types of antiretroviral regimen, side effects, and patients’ perception of risk and treatment benefits derived qualitatively further reinforced corresponding quantitative findings.

**Conclusion:**

Tailored socio-behavioral interventions are needed to address inherent differences within heterogeneous populations requesting nPEP, stigma, and patients’ perceptions of nPEP in order to improve follow-up and its adherence.

## Introduction

HIV/AIDS epidemics remain a growing public health challenge in Singapore and sexual transmission remains the dominant mode of spread. From when the first HIV/AIDS case was reported in 1985 through 2009, 65.9% of the cases were detected among heterosexuals, while 21.2% were reported among homosexuals and 6.8% among bisexuals.[1] Transmission pattern has however changed from heterosexual, constituting 45.1% of cases between 2010 and 2015, to increasingly homosexual and bisexual at 43.6% and 8.2% respectively.[1] HIV transmission can be effectively prevented using biomedical interventions. These include the correct and consistent use of condoms, antiretroviral therapy (ART) by people living with HIV and the use of pre and post-exposure prophylaxis. Non-occupational HIV post-exposure prophylaxis (nPEP) is a 28-day prescription of ART that is provided within 72 hours of an exposure to prevent an infection.[2–4]

Sexually transmitted infection (STI) management guidelines published by the Department of STI Control (DSC), Singapore, provide clear indications for nPEP, treatment regimens of choice, baseline tests, counseling points and follow-up criteria.[5] DSC guideline recommends a 28-day course of zidovudine/lamivudine (AZT/3TC), in combination with lopinavir/ritonavir (LPV/r), with HIV serostatus reassessed at 4 weeks, 3 months and 6 months post-exposure.[5] Between 2010 and 2016, AZT/3TC/LPV/r was offered by DSC Clinic at SGD$600 –$1000 (~USD$460 –$760). From November 2014 onwards, patients were given the option to purchase tenofovir disoproxil fumarate/emtricitabine (TDF/FTC) and ritonavir-boosted atazanavir (ATV/r) regimen from external sources (approximately SGD$100 –$200 (~USD$76 –$152)).

An audit conducted at the DSC clinic in 2010 recorded 66% medications adherence rate, 34% follow-up rate and 90% adherence to DSC guideline. However, associated factors affecting adherence and follow-up have yet to be comprehensively explored.[6] Outside Singapore, the presence of adverse events, high-risk sexual behavior, victims of sexual assault, and the use of AZT/3TC/LPV/r combination therapy have shown to correlate with decreased adherence.[7–10] Follow-up rates of nPEP patients were documented to be between 30–54% and the suboptimal follow-up rate has been associated with being a female, self-pay, non-consensual sexual exposure, and non-adherence to nPEP.[11, 12] Therefore, understanding the demographics of patients accessing nPEP, the accuracy of its prescriptions, as well as predictors of adherence and loss to follow-up (LTFU) may improve clinical practice and outcome.

This study aims to 1) describe characteristics of patients treated with nPEP, 2) identify predictors of LTFU and adherence to nPEP, and 3) to evaluate prescriptions against the current DSC STI management guideline. To further explain the results of the retrospective analysis, we qualitatively elicited healthcare staff perspectives on the barriers and facilitators to 1) follow-up, 2) adherence to nPEP and 3) adherence to DSC nPEP guideline.

## Methods

We used an explanatory sequential study design involving a retrospective cohort study, followed by in-depth interviews (IDI) with healthcare providers. The National Healthcare Group (NHG) Domain Specific Review Board (DSRB) approved the study. (NHG DSRB reference: 2016/00425). Waiver of consent was granted for this part of the study.

### Retrospective cohort study

#### Setting and study period

**Fig 1:**
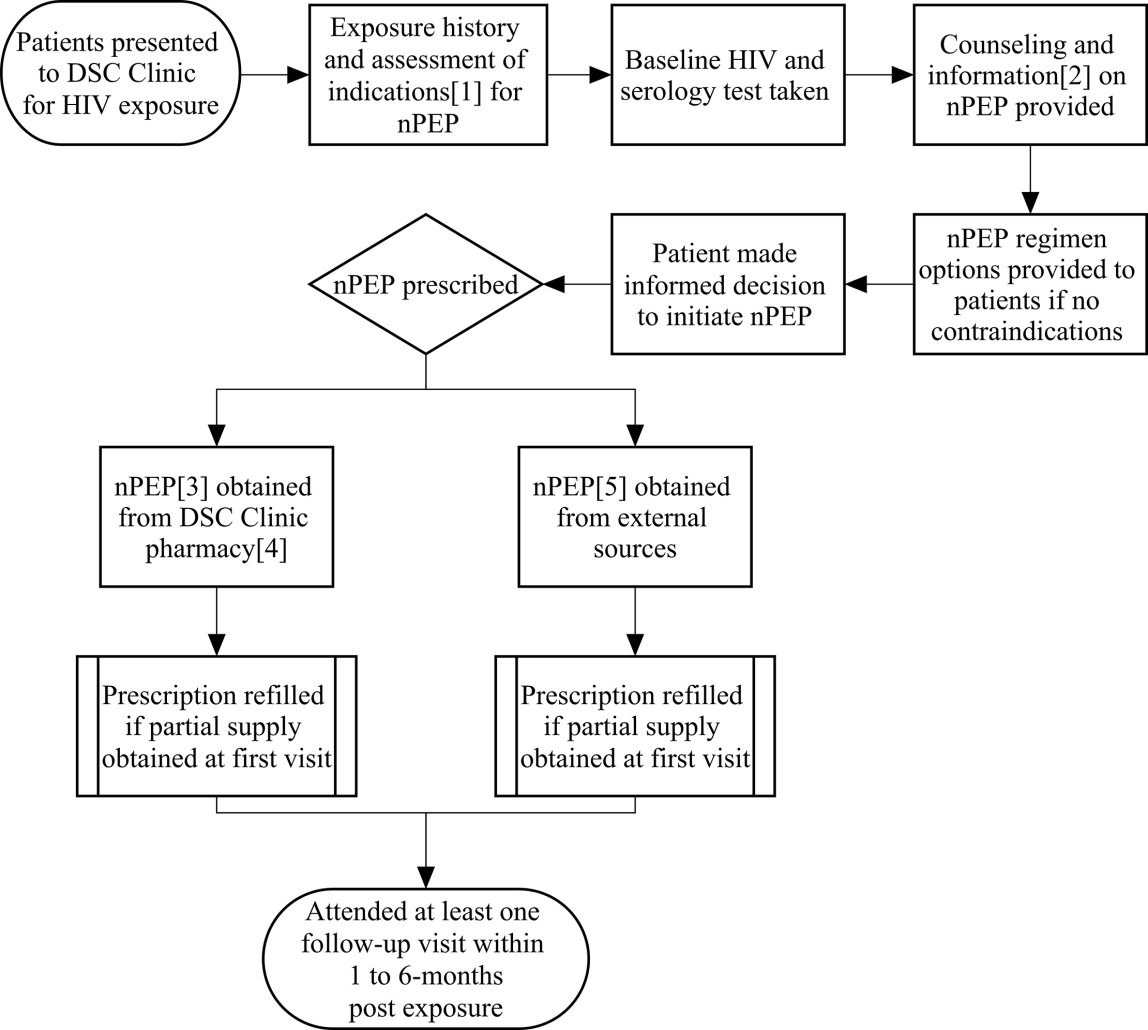
DSC guideline and nPEP flow. Patient flow through the Department of STI Control Clinic, Singapore for nPEP. 1) Indicated for high risk exposures that refer to condomless anal, vaginal intercourse or receptive oral sex with ejaculation with HIV positive partner, commercial sex workers, IV drug users, men who have sex with men, or was sexually assaulted. 2) Information on the risk of acquiring HIV, safe sex practices, risks and benefits of nPEP were provided. 3) nPEP regimen obtained from DSC Clinic pharmacy: AZT/3TC/LPV/r; zidovudine/lamivudine/lopinavir/ritonavir. 4) Patients had the option of purchasing any quantity of prescribed antiretroviral and refill the prescription thereafter. Most patients purchased 2-weeks supply at first visit. 5) nPEP regimen obtained from external sources: TDF/FTC/ATV/r; tenofovir/emtricitabine/atazanavir/ritonavir.

We conducted a retrospective analysis of all patients prescribed with nPEP (Fig 1) at the DSC Clinic between 1 January 2010 and 31 January 2016. De-identified demographics, clinical and prescription data were extracted from electronic medical and pharmacy records.

#### Variables

Demographic characteristics and the following variables were collected: time from exposure to baseline visit at DSC Clinic, source contact, HIV status of source contact, types of exposure, condom use during contact, nPEP regimen prescribed, dates of baseline and follow-up visits, information on counseling received, side effects, adherence to nPEP, repeat presenters and HIV test results.

#### Subjects

Patients were defined as at-risk for HIV if high-risk exposure—condomless anal, vaginal intercourse or receptive oral sex with ejaculation with HIV positive partner, commercial sex workers, IV drug users, men who have sex with men, or was sexually assaulted—was reported. Condomless sex (Condom use: no) was defined as condoms not used for any act of anal, vaginal and/or oral sex. Patients returning to DSC for nPEP following a new episode of exposure within the study period were considered separately. Repeat presenters were defined as having two or more requests for nPEP.

#### Baseline HIV status assessment

Baseline HIV status was ascertained using Determine® HIV-1/HIV-2 (Abbott) rapid test kit. Rapid test positive patients were required to undergo confirmatory testing by ELISA and Western Blot, and referrals were made to the Communicable Disease Centre, Tan Tock Seng Hospital Singapore for HIV care. [5]

#### Outcome measures and analysis

The characteristics of the study sample were described using frequencies and proportions. Categorical variables were analyzed using Chi-squared test and Fisher’s exact test. All statistical evaluations were made using STATA version 13 (Stata Corp, College Station, TX, USA).

**Loss to follow-up (LTFU):** Patients had scheduled follow-up at 4-weeks (post-treatment), 3 months and 6 months post-exposure. We considered LTFU if patients did not return to DSC Clinic for HIV test at least once within the follow-up schedule. Patients who returned within the first month to refill prescription and were seen by clinicians, but did not fulfill the post-treatment follow-up criterion were also considered LTFU.

**Adherence to nPEP:** Most patients received a combination therapy of AZT 300mg/3TC 150mg/ LPV 400mg/r 100mg twice daily or TDF 300mg/FTC 200mg/ATV 300mg/r 100mg once daily. Patients were followed-up at DSC clinic irrespective of the regimen they were prescribed. In addition, patients had the option of purchasing a full or partial supply of the prescribed course. Partially filled prescriptions were returned to patients for the remaining portions. Information on treatment completion was self-reported by the patients at subsequent follow-up. We defined patients who completed 28 days of treatment as adherent. The analysis was restricted to patients with documented treatment completion status.

Assessments of predictors associated with LTFU and adherence to nPEP were conducted using modified Poisson regression with robust sandwich variance. Variables achieving a p-value of ≤ 0.2 in the bivariate analyses were further considered for inclusion in the models. Risk ratios were reported with its associated 95% confidence interval to quantify the association.

**Adherence to nPEP guideline:** Prescription for nPEP were considered in accordance with the DSC 2007 and 2013 guidelines if it was prescribed to at-risk patients within 72 hours of exposure.

### In-depth interviews

#### Sampling and participant recruitment

Health care providers were purposively sampled in order to recruit high-value participants involved in nPEP management at the DSC Clinic. The Head of DSC Clinic and the co-investigator (MTWC) of this study acted as gatekeepers to potential participants. A letter of invitation including study background and objectives was sent via email to potential participants. Interviews were arranged with those who agreed to partake at a time and location of convenience. Written informed consent was taken and participation was voluntary. Participants were given a token of appreciation—Starbucks® gift card worth SGD$30 (~USD$23) at the end of the interview.

#### Data collection

In-depth interviews (IDI) were conducted from July to August 2016. All interviews (n = 9) were carried out in English by the principal investigator who is trained in qualitative research and conducted based on a semi-structured interview guide (S1 File) that was pilot tested prior to implementation. The guide comprised broader themes designed to understand barrier and facilitators to adherence to nPEP, follow-up, and guidelines implementation. Suggestions for improvements were also discussed. Each interview lasted 30 minutes and was audio recorded.

#### Analysis

All interviews were transcribed verbatim by the principal investigator. Annotation and analysis of complete transcripts were conducted using NVIVO 11 (QSR International). Textual references to topics of interest were retrieved and categorized using applied thematic analysis.[13] The initial themes based on main interview questions were used to develop a codebook of structural codes. Emergent codes were identified and added to the codebook. The principal investigator was the sole coder in this study. After an iterative process, the final codebook was applied to all interviews. Conclusions were drawn and verified through mapping, and interpretation of interview questions developed from text data set. Pre-existing and emerged themes were included. Qualitative data were synthesized with audit findings to give a complete understanding of nPEP management as a whole.

## Results

### Retrospective cohort study

#### Study participants’ characteristics

During the study period, 502 patients received nPEP at DSC Clinic. The cohort comprised heterosexuals (57%) and MSM/bisexual men (43%), with Singapore residents (75%) and men (97%) being the majority (Table 1). Condomless sex was higher in the MSM/bisexuals (72%) as compared to heterosexuals (53%). There were 26 (5.2%) repeat presenters in the study period.

**Table 1:** Patients demographics and exposure characteristics.

	Sexual orientation			*p-*value
	Heterosexual	MSM/Bisexual	Total	
	n = 284	n = 218	n = 502	
**Age group (years), n (%)**				0.060
<30	150 (52.8)	102 (46.8)	252 (50.2)	
30 to 39	112 (39.4)	84 (38.5)	196 (39.0)	
40 to 49	17 (6.0)	28 (12.8)	45 (9.0)	
≥ 50	5 (1.8)	4 (1.8)	9 (1.8)	
**Gender, n (%)**				0.001
Male	269 (94.7)	218 (100.0)	487 (97.0)	
Female	15 (5.3)	0 (0.0)	15 (3.0)	
**Ethnicity, n (%)**				<0.001
Chinese	172 (60.6)	169 (77.5)	341 (67.9)	
Malay	6 (2.1)	5 (2.3)	11 (2.2)	
Indians	46 (16.2)	4 (1.8)	50 (10.0)	
Others	60 (21.1)	40 (18.4)	100 (19.9)	
**Marital status**^**1**^**, n (%)**				<0.001*
Single	204 (71.8)	215 (98.6)	419 (83.5)	
Married	78 (27.4)	3 (1.4)	81 (16.1)	
Divorced	1 (0.4)	0 (0.0)	1 (0.2)	
**Residency status, n (%)**				0.090
Singapore residents	206 (72.5)	168 (77.1)	374 (74.5)	
Employment/student pass holders	52 (19.3)	41 (18.8)	93 (18.5)	
Tourists	26 (9.2)	9 (4.1)	35 (7.0)	
**Occupation, n (%)**				0.234
NS/SAF Regular/Office workers	32 (11.3)	22 (10.1)	54 (10.8)	
Professionals	129 (45.4)	82 (37.6)	211 (42.0)	
Technicians/Unskilled workers	43 (15.2)	45 (20.6)	88 (17.5)	
Student	35 (12.3)	37 (17.0)	72 (14.3)	
Unemployed	10 (3.5)	10 (4.6)	20 (4.0)	
Others	35 (12.3)	22 (10.1)	57 (11.4)	
**Source contact, n (%)**				<0.001*
Regular	4 (1.4)	20 (9.2)	24 (4.8)	
Casual	107 (37.7)	183 (83.9)	290 (57.8)	
Commercial sex workers	168 (59.2)	13 (5.9)	181 (36.0)	
Sexual assault	4 (1.4)	1 (0.5)	5 (1.0)	
Non-sexual/others	1 (0.3)	1 (0.5)	2 (0.4)	
**Source contact's HIV status, n (%)**				<0.001*
Negative	1 (0.4)	1 (0.5)	2 (0.4)	
Positive	6 (2.1)	28 (12.8)	34 (6.8)	
Unknown	277 (97.5)	189 (86.7)	466 (92.8)	
**Exposure type, n (%)**				
Insertive vaginal	250 (88.0)	3 (1.4)	253 (50.4)	<0.001*
Receptive vaginal	15 (5.3)	0 (0.0)	15 (3.0)	<0.001*
Insertive anal	7 (2.5)	87 (40.0)	94 (19.7)	<0.001
Receptive anal	2 (0.7)	130 (59.6)	132 (26.3)	<0.001*
Unsure anal position	0 (0.0)	1 (0.5)	1 (0.2)	0.434*
Receptive/insertive oral	125 (44.0)	119 (54.6)	244 (48.6)	0.019
Unsure anal/vaginal	2 (0.7)	0 (0.0)	2 (0.4)	0.508*
IV drug use	0 (0.0)	1 (0.5)	1 (0.2)	0.434*
Others	8 (2.8)	6 (2.8)	14 (2.8)	0.595
**Condom use**^**2**^^,^^**3**^^,^^**4**^**, n (%)**				<0.001*
Yes	7 (2.5)	5 (2.3)	12 (2.4)	
No	150 (52.8)	156 (71.6)	306 (60.9)	
Torn/slipped	114 (40.1)	48 (22.0)	162 (32.3)	
Unsure	3 (1.1)	1 (0.5)	4 (0.8)	
**Risk categories, n (%)**				0.521
At risk	246 (86.6)	193 (88.5)	439 (87.5)	
Low/not warranted	38 (13.4)	25 (11.5)	63 (12.5)	

nPEP, non-occupational post-exposure prophylaxis; MSM, men who have sex with men; IV, intravenous; NS, national servicemen; SAF, Singapore Armed Forces

^1^One observation with undocumented status (heterosexual, 1)

^2^Four observations with undocumented condom use (heterosexual, 2; MSM/bisexual, 2)

^3^Fourteen observations where condom use was not applicable (heterosexual, 8; MSM/bisexual, 6)

^4^Condom use: Used for every act of anal, vaginal and/or oral sex. Condom not used/condomless sex was defined as not using condoms for any one act of anal, vaginal and/or oral sex.

*Fisher’s exact test

#### Loss to follow-up

Of the 502 observations, one patient was tested positive at baseline using Determine® HIV-1/HIV-2 (Abbott) rapid test kit (Table 2). nPEP was prescribed in view of recent seroconversion. This patient remained HIV positive after 2 weeks of nPEP and a referral to the Communicable Disease Centre was made. No seroconversions were otherwise documented amongst those who were followed-up. There were 230 (46%) observations who were LTFU during the study period. Age, gender, residency status, and sexual orientation were adjusted in the final multivariate model. Residency status, in particular, tourists (aRR, 2.29 [1.90–2.74]; *p*<0.001) and being MSM/bisexuals (aRR, 1.32 [1.09–1.59]; *p* = 0.004) were significantly associated with LTFU (Table 3).

**Table 2:** Intervention, follow-up, and adherence to nPEP.

	Sexual orientation			*p-*value
	Heterosexual	MSM/Bisexual	Total	
	n = 284	n = 218	n = 502	
**Exposure to nPEP time**^**1**^**, n (%)**				0.328*
Less than 24 hours	75 (26.4)	71 (32.6)	146 (29.1)	
24 to 47 hours	91 (32.0)	73 (33.5)	164 (32.7)	
48 to 72 hours	113 (39.8)	69 (31.7)	182 (36.2)	
More than 72 hours	4 (1.4)	4 (1.8)	8 (1.6)	
Not documented	1 (0.4)	1 (0.5)	2 (0.4)	
**Types of ARV prescribed, n (%)**				<0.001
AZT/3TC/LPV/r	248 (87.3)	152 (69.7)	400 (79.7)	
TDF/FTC/ATV/r	36 (12.7)	66 (30.3)	102 (20.3)	
**nPEP side effects**^**2**^^,^^**3**^**, n (%)**				0.392
Yes	188 (83.6)	138 (80.2)	326 (82.1)	
No	37 (16.4)	34 (19.8)	71 (17.9)	
**Baseline HIV tests results**^**4**^**, n (%)**				0.537*
Yes, negative	276 (97.2)	212 (97.2)	488 (97.2)	
Yes, positive	0 (0.0)	1 (0.5)	1 (0.2)	
Tests not done	7 (2.5)	4 (1.8)	11 (2.2)	
**Follow-up HIV test results**^**5**^**, n (%)**				0.390*
Negative	169 (59.5)	107 (49.1)	276 (55.0)	
Positive	0 (0.0)	1 (0.5)	1 (0.2)	
**nPEP repeat presenters, n (%)**				0.012*
Twice	8 (2.8)	16 (7.3)	24 (4.8)	
More than twice	0 (0.0)	2 (0.9)	2 (0.4)	
None	276 (97.2)	200 (91.7)	476 (94.8)	
**Adherence to nPEP**^**6**^**, n (%)**				0.613
Adherent (completed treatment)	132 (73.7)	78 (76.5)	210 (74.7)	
Did not complete treatment	47 (26.3)	24 (23.5)	71 (25.3)	
**Follow up (at least once within 1 to 6 months post-exposure), n (%)**				0.018
Yes	167 (58.8)	105 (48.2)	272 (54.2)	
No	117 (41.2)	113 (51.8)	230 (45.8)	

nPEP, non-occupational post-exposure prophylaxis; ARV, antiretroviral; MSM, men who have sex with men antiretroviral; AZT/3TC/LPV/r, zidovudine/lamivudine/lopinavir/ritonavir; TDF/FTC/ATV/r, tenofovir/emtricitabine/atazanavir/ritonavir.

^1^Two observations with undocumented time between exposure and prescription of NPEP (Heterosexual, 1; MSM/bisexual, 1)

^2^Ninety three observations did not return to the clinic after the first visit (Heterosexual, 53; MSM/bisexual, 40)

^3^Twelve observations with undocumented presence or absence of side effects (Heterosexual, 6; MSM/bisexual, 6)

^4^Two observations with undocumented baseline HIV tests results (Heterosexual, 1; MSM/bisexual, 1)

^5^Two hundred and twenty-five observations with unknown HIV results (Heterosexual, 115; MSM/bisexual, 110)

^6^Two hundred and twenty one observations with undocumented treatment completion or non-completion (Heterosexual, 105; MSM/bisexual 116)

*Fisher’s exact test

**Table 3:** Predictors of loss to follow-up.

Loss to follow up	Crude RR (95% CI)	*p-*value	Adjusted RR (95% CI)	*p-*value
**Age group (years)**		0.887*		0.935*
<30	1		1	
30 to 39	1.00 (0.82–1.23)	0.971	0.98 (0.80–1.20)	0.855
40 to 49	1.08 (0.78–1.50)	0.643	0.99 (0.72–1.35)	0.929
≥ 50	1.23 (0.67–2.24)	0.503	1.19 (0.67–2.12)	0.560
**Gender**				
Male	1		1	
Female	0.72 (0.35–1.49)	0.376	0.78 (0.41–1.49)	0.451
**Residency status**		<0.001*		<0.001*
Singapore residents	1		1	
Employment/student pass holders	1.18 (0.92–1.50)	0.192	1.18 (0.93–1.50)	0.184
Tourist	2.15 (1.81–2.55)	<0.001	2.29 (1.90–2.74)	<0.001
**Sexual orientation**				
Heterosexual	1		1	
MSM/bisexual	1.26 (1.04–1.52)	0.017	1.32 (1.09–1.59)	0.004

CI, confidence interval; RR, risk ratio; MSM, men who have sex with men.

*Overall *p*-value

#### Adherence to nPEP

Among the 281 patients (56%) with documented treatment completion or non-completion, 50 patients (90%) and 160 (71%) completed 28-days of TDF/FTC/ATV/r and AZT/3TC/LPV/r, respectively. Side effects were reported in 326 (65%) cases, with diarrhea, nausea, fatigue, and rash being the most commonly stated. Forty (8%) cases had raised liver function tests (Table 4). Thirty-four patients discontinued treatment due to side effects and four stopped treatment because their partner was tested negative. Thirty-three patients defaulted treatment without specific reasons. After accounting for age, gender, residency status, and sexual orientation, TDF/FTC/ATV/r regimen (aRR, 1.15 [1.03–1.29]; *p* = 0.017), and absence of side effects (aRR, 1.14 [1.02–1.27]; *p* = 0.024) were positively associated with adherence (Table 5).

**Table 4:** Side effects according to antiretroviral regimen prescribed.

Side effects	nPEP regimens		*p*-value
	AZT/3TC/LPV/r n = 400	TDF/FTC/ATV/r n = 102	
**Gastrointestinal, n (%)**			
Nausea	121 (30.3)	12 (11.8)	<0.001
Metallic taste	5 (1.3)	0 (0.0)	0.588*
Loss of appetite	17 (4.3)	1 (1.0)	0.142*
Dry mouth	0 (0.0)	1 (1.0)	0.203*
Diarrhea	170 (42.5)	14 (13.7)	<0.001
Vomiting	8 (2.0)	2 (2.0)	1.000*
Abdominal pain	26 (6.5)	1 (1.0)	0.025*
**Dermatologic, n (%)**			
Rash	29 (7.3)	5 (4.9)	0.400
**Hepatic, n (%)**			
Elevated ALT/AST	10 (2.5)	3 (2.9)	0.733*
Elevated bilirubin	1 (0.3)	26 (25.5)	<0.001*
**Neurologic, n (%)**			
Headache	11 (2.8)	1 (1.0)	0.474*
Insomnia	3 (0.8)	1 (1.0)	1.000*
Dizziness	7 (1.8)	1 (1.0)	1.000*
**Respiratory, n (%)**			
Cough	0 (0.0)	1 (1.0)	0.203*
**Musculoskeletal pain, n (%)**	5 (1.3)	0 (0.0)	0.588*
Fatigue, n (%)	64 (16.0)	8 (7.8)	0.036
Pedal edema, n (%)	2 (0.5)	0 (0.0)	1.000*
Missing data, n (%)	80 (20.0)	25 (24.5)	

nPEP, non-occupational post exposure prophylaxis; ALT, alanine transaminase AST, aspartate transaminase; AZT/3TC/LPV/r, zidovudine/lamivudine/lopinavir/ritonavir; TDF//FTC/ATV/r

tenofovir/emtricitabine/atazanavir/ritonavir

*Fisher exact test

**Table 5:** Predictors of adherence to nPEP.

Adherence (treatment completion)	Crude RR (95% CI)	*p-*value	Adjusted RR^1^ (95% CI)	*p-*value
**Age group (years)**		0.403*		0.557*
<30	1		1	
30 to 39	0.96 (0.82–1.11)	0.583	0.93 (0.81–1.06)	0.272
40 to 49	1.11 (0.91–1.35)	0.292	0.99 (0.82–1.20)	0.898
≥ 50	0.66 (0.29–1.48)	0.314	0.71 (0.36–1.39)	0.319
**Gender**				
Male	1		1	
Female	1.21 (0.98–1.51)	0.083	1.22 (1.09–1.37)	<0.001
**Residency status**		0.824*		0.165*
Singapore residents	1		1	
Employment/student pass holders	1.04 (0.88–1.23)	0.635	1.08 (0.94–1.24)	0.292
Tourist	0.90 (0.51–1.59)	0.709	1.20 (0.98–1.48)	0.085
**Sexual orientation**				
Heterosexual	1		1	
MSM/bisexual	1.04 (0.90–1.19)	0.608	1.03 (0.91–1.17)	0.645
**Types of ARV prescribed**				
AZT/3TC/LPV/r	1		1	
TDF/FTC/ATV/r	1.28 (1.14–1.45)	<0.001	1.15 (1.03–1.29)	0.017
**Side effects**				
Yes	1		1	
No	1.17 (1.05, 1.31)	0.004	1.14 (1.02–1.27)	0.024

CI, confidence interval; RR: risk ratio; nPEP, non-occupational post exposure prophylaxis; MSM, men who have sex with men; ARV, antiretroviral; AZT/3TC/LPV/r, zidovudine/lamivudine/lopinavir/ritonavir; TDF/FTC/ATV/r, tenofovir/emtricitabine/atazanavir/ritonavir

*Overall *p*-value

#### Adherence to guideline

There were 439 (88%) nPEP patients who were classified as at-risk and 492 (98%) of them received nPEP within 72 hours of exposure. All patients were thoroughly counseled and made informed decisions to initiate nPEP. Baseline HIV status of 488 patients (97%) were ascertained negative prior to nPEP initiation. Prescription in accordance with DSC guidelines was made for 431 (86%) patients.

### In-depth interviews

#### Study participants’ characteristics

Nine IDIs were conducted at the participants’ workplace. The median age was 31 years (IQR 29–34). Other characteristics are summarized in Table 6.

**Table 6:** Characteristics of participants at qualitative in-depth interview.

	n = 9	%
**Age in years, median (IQR)**	31 (29–34)	
**Gender**		
Male	5	55.6
Female	4	44.4
**Ethnicity**		
Chinese	7	77.8
Malay	2	22.2
**Position at DSC Clinic**		
Nurse	3	33.3
Pharmacy technician	1	11.1
Counselor	1	11.1
Doctor	4	44.4
**Years of experience, median (IQR)**		
At DSC Clinic	3 (2–7)	
Working with HIV	3 (3–5)	

IQR, Interquartile Range; HIV, Human immunodeficiency virus

#### Barriers and facilitators to follow-up

Availabilities of other HIV testing sites and avenues for subsequent follow-up were factors identified as barriers for patients to return to DSC Clinic for follow-up. That includes anonymous testing sites, which were specifically indicated by some patients as their avenue of preference.

“*They might not come back for the repeat test because there are a lot of other avenues where they can get their HIV test done anonymously*” *(ID303)*

Some participants expressed the problem of patients having misconceptions about the benefits of medications and therefore defaulting subsequent consultations and follow-ups. All participants who brought up this issue mentioned patients’ confidence that medications would work and thus defaulted follow-ups. Anxious patients were more likely to return for HIV testing after treatment completion.

“*And many of them felt that even you take PEP, they have this mindset that PEP does have a 100% coverage. That’s why they don't come back for further follow-up*” *(ID201)*

Counseling and advice given by doctors for patients to adhere to follow-up schedule were identified as one of the driving factors. Information about potential side effects of medications provided during mandatory counseling session encouraged patients to return for follow-up. Participants highlighted the importance of a reminder system as an active prompt to improve follow-up rate.

“*Once we inform about the side effect of the medication, so most of them do come back for the follow-up. Just to confirm whether they get any bad side effect from the medication*” *(ID103)*

#### Barriers and facilitators to adherence to nPEP

Most participants expressed that intolerable side effects were the major contributor to medications non-adherence.

“*Of course, many times they gave up because of the ill effects*” *(ID401)*

The high cost of medications was one of the main factors identified as a deterrent for refilling of prescription and completing the course.

“*I mean I am aware of the cost issues. So that could be another major factor why they, you know stop being compliant to the PEP*” *(ID304)*

Low perception of threat and risk of contracting HIV was another barrier to adherence. The perception of threat dissipated after a short while and thus decreased patients’ motivation to continue with the course. Self-perceived chances of contracting HIV and severity of the situation otherwise facilitated treatment adherence.

“*Maybe they felt with time, their risk was less. Other than that, I mean, I suppose they just felt that the problem wasn’t big enough for them to continue the medications*” *(ID304)*

#### Barriers and facilitators to adherence to clinical guideline

Patients’ insistence was a major barrier to guideline adherence.

“*At times, whereby they are really worried, even though doctor decline their request, they will just return the next day within 72 hours just to return and beg for us to let him see the doctor*” *(ID101)*

Most participants agreed that the DSC STI guidelines were clear, easy to follow and furnished with important information. Opportunities to consult senior clinicians were highlighted as a facilitator as well.

“*I think the guidelines are quite clear. So most of us actually follow the guidelines first. And we do have to clear with the senior doctor before we prescribe*” *(ID302)*

#### Under-representation of women

Under-representation of women was one of the emerged themes. Participants explained that this phenomenon could be due to key populations disproportionately at risk of HIV, for instance, sex workers’ clientele and MSM were predominantly men. However, some women might be at risk but they were under-represented due to stigma and the society is conservative.

“*Females, on the whole, are lower risk group. And possibly because our society is still fairly conservative*” *(ID304)*

#### Over-reliance on nPEP

Most participants mentioned that complacency was the major factor behind repeat presenters. However, some participants perceived that this was not a major issue due to proper counseling and complexity of the course. Nevertheless, they agreed that special attention should be given to this population.

“*If they come in for the 2nd time that also means that they have not accepted the message of practicing safe sex, for whatever reasons. I think we have to pay more attention to this group*” *(ID301)*

## Discussion

Non-occupational post-exposure prophylaxis is an imperative component of public health strategy for HIV prevention in Singapore. Using five years of clinical data, we described nPEP practices at DSC Clinic, Singapore. Most patients prescribed with nPEP at DSC clinic were young men potentially exposed to HIV through high-risk sexual behavior; a trend observed in other settings.[14–17] Among the 502 observations, only 15 were females. Under-representation of women was one of the emerged qualitative themes where disproportionate risk, stigma, and conservatism highlighted by interviewees substantiated this finding. Repeat presenters constituted 5% of the sampled population; however, 65% of them were MSM/bisexual. The predominance of MSM/bisexual as nPEP repeat presenters were also observed in other studies.[17, 18] Despite the inverse association between previous nPEP use and increased risky sexual behavior among high-risk MSM, [19] special attention should be given to the repeat presenters for risk compensation, including the provision of risk behavior counseling and considerations for pre-exposure prophylaxis (PrEP) as an alternative prevention tool. PrEP was introduced in Singapore in 2016 and it is currently available at several public and private healthcare institutions. It is recommended for individuals in serodiscordant relationships, PEP repeat presenters, and at-risk groups such as MSM, intravenous drug users, and those who are not in a mutually monogamous relationship.

### Loss to follow-up

HIV-testing follow-up remained low (54%), as observed in other similar studies.[4, 12, 20] This could be ascribed to the availability of other avenues (anonymous test sites, private clinics, and hospitals) to get a follow-up HIV test. In this study, MSM was significantly associated with loss to follow-up. Although criminalization of homosexuality in Singapore is not proactively enforced,[21] the proscription hinders MSM to undertake follow-up HIV tests at a denominated center. Preference for anonymous HIV-testing over sequential testing at DSC Clinic is further fortified by consequences of reporting HIV status to health authorities, and pertinence of HIV stigma and discrimination locally.[22, 23] Nevertheless, the follow-up rate has improved from a previous audit (34%)[6] conducted in 2010 and this could be attributed to a larger sample size and a higher proportion of residents, employment and student pass holders (resides in Singapore for an extended period) who were prescribed with nPEP in this study. The misconception about a treatment’s efficacy was identified by IDI participants as a barrier and this underscored the importance of counseling and education. As for ways to improve follow-up rate, IDI participants expressed the importance of setting up an active reminder system [7, 20, 24] and this intervention could be implemented locally.

### Adherence to nPEP

Our result showed relatively discouraging data on nPEP adherence.[4, 7, 8, 12, 25] Tourists and MSM/bisexuals were found to be at higher risk of LTFU and this correspondingly, affected how adherence was documented and assessed. This is consistent with a study in the UK which associated poor adherence to high default rate from follow-up and poor documentation.[20] In our study, patients treated with TDF/FTC/ATV/r were 15% more likely to adhere to the course in comparison to AZT/3TC/LPV/r and this association is in agreement with other studies.[8, 9, 16, 26] Adherence to treatment remained higher in patients treated with TDF/FTC/ATV/r after accounting for side effects in the model. This could be attributed to other factors such as lower pill burden (once daily vs. twice daily) and the higher proportion of patients who could afford to purchase the entire treatment course of TDF/FTC/ATV/r (32%) after the first nPEP consultation compared to AZT/3TC/LPV/r (11%). Insights from IDI participants highlighted that side effects were important considerations affecting nPEP adherence, and this further supported our quantitative findings. TDF/FTC is also the World Health Organization (WHO) recommended backbone antiretroviral (ARV) regimen for HIV PEP in adults and adolescents.[27] Therefore, TDF/FTC/ATV/r that is tolerable, with simplified once-daily dosing and more affordable ought to be considered as HIV nPEP standard of care.

### Adherence to nPEP guideline

Out of 502 prescriptions, 86% were prescribed accurately in accordance with DSC guidelines. This result is comparable to guideline adherence rate in other centers, including the 2010 audit at the DSC Clinic.[4, 6, 17] Availability of clear guideline and opportunities to consult senior clinicians were two facilitators of guideline adherence identified by IDI participants and they are in agreement with a study published in Belgium where nPEP prescriptions were found to be 99% accurate.[17] IDI participants also emphasized patients’ demand as a barrier to guideline adherence. Clinicians faced with anxious patients demanding for nPEP often prescribe it despite low-risk exposures highlighted challenges in caring for potentially HIV-exposed patients.[4, 28]

The strengths of this study include the large number of cases reviewed, relative to the 2010 audit. Comprehensive information of nPEP patients, source contact and exposures were extracted and analyzed in this study. To our knowledge, this is the first study to explore predictors of adherence and LTFU in Singapore, and therefore embodies potential programmatic decision implications. Adoption of explanatory sequential design in this mixed method study allowed the inclusion of providers’ perspectives to better comprehend quantitative findings. Multiple viewpoints from high-value subjects provided an understanding of complex situations and behavior of nPEP patients and practices in Singapore. Utilization of a semi-structured interview guide for all IDI ensures consistency and only one coder was involved in the analysis of qualitative data, thus facilitating an in-depth understanding of the data. The validity of findings is further strengthened through the triangulation of both quantitative and qualitative data.

The findings of this study must be deliberated in the context of several weaknesses. Quantitative data were collected retrospectively from a single healthcare institution. Hence, study findings cannot be extrapolated to all nPEP patients in Singapore. Secondly, data on source contact, their HIV status, adherence to nPEP and symptoms of side effects were self-reported by patients during the consultation, subjected to self-reporting bias. Adherence documentation was associated with follow-up and might be underestimated due to low follow-up rate. Information on nPEP refusals was not captured, as pharmacy records were the primary source to extract nPEP patients’ identifiers. Qualitatively, the patients’ perspectives were not elicited and the applicability of qualitative findings are limited to the providers at DSC Clinic. Due to limited sample size, data saturation for all topics was not achieved with the exception of barriers and facilitators of adherence to medications.

## Conclusion

The results of this study highlighted the need to look into the barriers to follow up and treatment adherence to enhance the efficacy of nPEP. Our study shows that types of regimen and MSM are associated with adherence and poor follow-up rate. Therefore, it is important to acknowledge the diversity of population accessing nPEP and the subpopulation who are disproportionately at risk of loss to follow-up. Tailored socio-behavioral interventions are needed to address the inherent differences within heterogeneous populations requesting nPEP, underlying stigma and patients’ perception of nPEP in order to improve follow-up and treatment adherence.

## Supporting information

S1 File Semi-structured interview guide. (PDF)

S2 File STROBE statement. (DOC)

S3 File COREQ checklist for interviews and focus groups. (PDF)
