# Supplementary material for: A mixed methods study of non-occupational post-exposure prophylaxis at an STI clinic in Singapore: Five-year retrospective analysis and providers' perspectives
Source: PLoS One. 2018 Aug 20;13(8):e0202267. doi: 10.1371/journal.pone.0202267 (PMC6101390; doi:10.1371/journal.pone.0202267)
Supplement: S1 File — (PDF) [file pone.0202267.s001.pdf]

In-Depth Interview (IDI) Guide
Target Audience: Non-occupational post exposure prophylaxis (NO-PEP) care providers at DSC Clinic, Singapore.

Topics
1. Barriers to patients' uptake and adherence to NO-PEP
2. Facilitators to patients' uptake and adherence to NO-PEP
3. Barriers to implementation of NO-PEP guidelines
4. Facilitators to implementation of NO-PEP guidelines

Draft (Additional probes and questions to be identified after audit analysis and field testing the interview guide)

1. Warm-up

1.1. Can you describe your role in the management of NO-PEP patients?
1.2. What do you think are the roles of NO-PEP in HIV prevention?
1.3. Can you describe your experience with the last occasion of a patient needed NO-PEP?

Supplementary warm up questions
Based on clinic's data, there were X% of patients on NO-PEP regimen for the last 5 years. With reference to risk categories, what kind of patients do you see most?

What do you think of HIV/AIDS situation in Singapore?

Now we would like to talk about successful treatment experiences in treating patients with NO-PEP, starting with the first part on patients' uptake and adherence.

2. Facilitators to patient uptake and adherence, and guidelines implementation

NO-PEP uptake rate from 2009-2015 was X%. Why do you think the rate was good?

Can you describe your experience with a patient with very good adherence from the beginning till the end? (From data: X% are compliant)

2.1. From your perspective, why do you think it was a successful case?
2.1.1.1. In this case, what do you think helped that patient adhere to NO-PEP
2.1.1.2. What were the factors that facilitated his/her action? (E.g. prior knowledge, peers/family support, awareness campaigns)

Next we would like to ask you to reflect on your experiences/colleague experiences being on the provider side

2.2. Referring to the scenario above, can you describe your contribution to treatment success of that patient?
2.3. Can you explain scenarios where guideline implementation went smoothly?
2.3.1. In your opinion, how do you think these positive outcomes can be sustained in the long run?

Moving on, we would like to talk about scenario where patients' uptake and compliance is suboptimal.

3. Barriers to patient uptake and adherence, and guidelines implementation

Despite a good uptake rate (*assuming from data)*, only X% of patients were able to comply and complete the full course.
If uptake rate is poor: NO-PEP uptake rate from 2009-2015 was X%. Why do you think the rate was good/bad?

Can you describe your experience with a patient with poor adherence from the beginning till the end?

- 3.1. Why is this patient's adherence poor?
  - 3.1.1. How different do you think is their perception of susceptibility and severity to HIV as compared to patients who were compliant?
  - 3.1.2. What do you think are their level of self efficacy (belief in one's ability to accomplish a task) to adhere to prescribed treatments?
  - 3.1.3. What do you think are the barriers to uptake and adherence? (access e.g. geographical location, cost of treatment, stigma, criminalization of MSM; section 377A)

Next, we would like to ask you to reflect on your experiences/colleague experiences being on the provider side

- 3.2. Can you explain scenarios where guideline implementation was met with obstacles and problematic?
  - 3.2.1. In your opinion, how do you think they can be improved?

4. How do you think we can improve NO-PEP uptake and compliance rate?

- 4.1.1.1. What do you think of the general awareness of NO-PEP and how can we improve it? Do you think the current education and counseling protocol is sufficient to improve compliance and prevent future exposure? (follow up: active recall)
- 4.1.1.2. What do you think of public's access to NO-PEP? (costs, stigma, location, services, decentralize clinical management to polyclinics/GPs)
- 4.1.1.3. What do you think of the current training program specifically to NO-PEP management? (if compliance to guidelines is suboptimal)
- 4.1.1.4. How can we improve referrals from other institutions? (referrals process)

5. If you are to compare between success and failure cases, what did the patient do differently?

6. Lastly, do you have anything to add that we have not spoken about?
